# Remimazolam: Non-Clinical and Clinical Profile of a New Sedative/Anesthetic Agent

**DOI:** 10.3389/fphar.2021.690875

**Published:** 2021-07-20

**Authors:** Gavin J. Kilpatrick

**Affiliations:** GJK Pharma Ltd., Eltisley, United Kingdom

**Keywords:** remimazolam, sedation, anesthesia, benzodiazepine, midazolam, propofol, total intravenous anesthesia, short-acting anesthetic

## Abstract

A program to identify novel intravenous sedatives with a short and predictable duration of action was initiated in the late 1990’s by Glaxo Wellcome. The program focussed on the identification of ester-based benzodiazepine derivatives that are rapidly broken down by esterases. Remimazolam was identified as one of the lead compounds. The project at Glaxo was shelved for strategic reasons at the late lead optimization stage. Via the GSK ventures initiative, the program was acquired by the small biotechnology company, TheraSci, and, through successive acquisitions, developed as the besylate salt at CeNeS and PAION. The development of remimazolam besylate has been slow by industry standards, primarily because of the resource limitations of these small companies. It has, however, recently been approved for anesthesia in Japan and South Korea, procedural sedation in the United States, China, and Europe, and for compassionate use in intensive care unit sedation in Belgium. A second development program of remimazolam was later initiated in China, using a slightly different salt form, remimazolam tosylate. This salt form of the compound has also recently been approved for procedural sedation in China. Remimazolam has the pharmacological profile of a classical benzodiazepine, such as midazolam, but is differentiated from other intravenous benzodiazepines by its rapid conversion to an inactive metabolite resulting in a short onset/offset profile. It is differentiated from other intravenous hypnotic agents, such as propofol, by its low liability for cardiovascular depression, respiratory depression, and injection pain. The benzodiazepine antagonist flumazenil can reverse the effects of remimazolam in case of adverse events and further shorten recovery times. The aim of this review is to provide an analysis of, and perspective on, published non-clinical and clinical information on 1) the pharmacology, metabolism, pharmacokinetics, and pharmacodynamic profile of remimazolam, 2) the profile of remimazolam compared with established agents, 3) gaps in the current understanding of remimazolam, 4) the compound’s discovery and development process and 5) likely future developments in the clinical use of remimazolam.

## Introduction

### Intravenous Benzodiazepines

The first benzodiazepine with “tranquilizing” properties, chlordiazepoxide, was synthesized in the 1950’s (Sternbach, 1979). Its discovery led to a large number of derivatives, including many compounds that have been developed and marketed as drugs. Benzodiazepines bind to an extracellular site at the interface of the α and γ subunits of the gamma-aminobutyric acid type A (GABA_A_) receptor to modulate the activity of the brain’s major inhibitory neurotransmitter, GABA (Sigel and Ernst, 2018; Kim and Hibbs, 2021). Most benzodiazepines induce a positive allosteric modulation of this ligand-gated chloride ion channel, resulting (usually) in neuronal hyperpolarization and inhibition of activity. Compounds acting at this site have been widely used as anxiolytics and sedatives and to induce muscle relaxation, amnesia, sleep, and anticonvulsive effects (Haefely et al., 1993).

**FIGURE 1:**
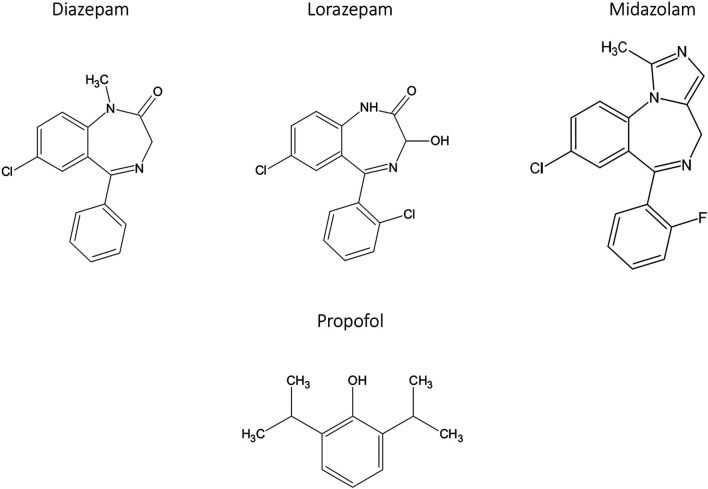
Intravenous benzodiazepines and propofol.

The low aqueous solubility of most benzodiazepines means that intravenous formulations are problematic. Nevertheless, intravenous formulations of several benzodiazepines, including diazepam and lorazepam (Figure 1), have been developed for procedural sedation and anesthesia (Cornett et al., 2018); however, they have a long duration of action and can cause injection pain and thrombosis at the site of injection (Hegarty and Dundee, 1977). A significant development in the field of intravenous benzodiazepines was midazolam’s introduction in 1982 as a water-soluble benzodiazepine (Figure 1) with a short duration of action (Dundee et al., 1984; Kanto, 1985). Midazolam is used widely as an intravenous agent to provide sedation for diagnostic and interventional procedures and longer-term sedation, including intensive care. However, midazolam has shortcomings. Most importantly, recovery is relatively slow and can be extended due to metabolism by cytochrome P450 3A4 and the production of active metabolites (Bauer et al., 1995). The dependence on cytochrome P450 3A4 means that drug-drug interactions can be problematic since many drugs inhibit this enzyme (Yuan, Flockhart and Balian, 1999).

### Propofol

Propofol (Figure 1) was discovered by ICI (now part of Astra Zeneca) (Glen and Hunter, 1984) and approved for the induction and maintenance of anesthesia in 1986. The precise mechanism of action of propofol is not fully understood, but it is thought to mediate much of its effects by positive modulation of the GABA_A_ receptor. This is the same receptor where benzodiazepines act, but propofol is thought to act at a different site, the β_3_ subunit (Jurd et al., 2003). It is not soluble in water and is formulated as a lipid-based emulsion. Propofol is the most widely used intravenous anesthetic drug globally and has revolutionized anesthetic practice and significantly improved patient care (Wood and Stark, 2018). Propofol has a very fast onset and offset of action. The short offset results from the redistribution into lipid compartments rather than rapid metabolism (Simons et al., 1988).

The use of propofol is usually restricted to anesthetists because of its potential to induce profound sedative effects associated with hypotension, bradycardia, respiratory depression, and potentially apnea (Perel, 2011; Sahinovic et al., 2018). Other adverse events related to propofol include pain on injection, which can be severe (Tan and Onsiong, 1998). The local anesthetic lignocaine is often co- or pre-administered to mitigate this (Kamal et al., 2021). The pain on injection induced by propofol is thought to be mediated by off-target interaction with the transient receptor potential receptors, TRPA1 and TRPV1, on sensory neurons (Cornett et al., 2008; Matta et al., 2008). Prolonged propofol infusion at high doses is also associated with a rare but potentially fatal syndrome termed propofol infusion syndrome (PRIS) (Bray, 1998; Hemphill et al., 2019).

### Ester-Based Short-Acting Drugs

Short-acting drugs provide the advantage of predictable control of their effects. Drugs with ester groups that are rapidly broken down to inactive acid metabolites have been used widely in anesthesia (Birgenheier et al., 2020) to provide controllable sedation/anesthesia, neuromuscular blockade, analgesia, and to regulate heart rate. These include the short-acting opiate analgesic, remifentanil (Feldman et al., 1991), neuromuscular blockers such as mivacurium (Savarese et al., 1988), and the beta_1_ blockers esmolol (Erhardt et al., 1982) and landiolol (Iguchi et al., 1992). Further discovery programs of intravenous ester-based anesthetic short-acting drugs have been reported more recently, including the topic of this review, remimazolam, the propanidid derivative AZD-3043 (Egan et al., 2012), and cyclopropyl methoxycarbonyl metomidate (AB700) (Ge et al., 2012). The latter two compounds do not appear to have progressed beyond early clinical trials.

### The Short-Acting Benzodiazepine Discovery Project

**FIGURE 2:**
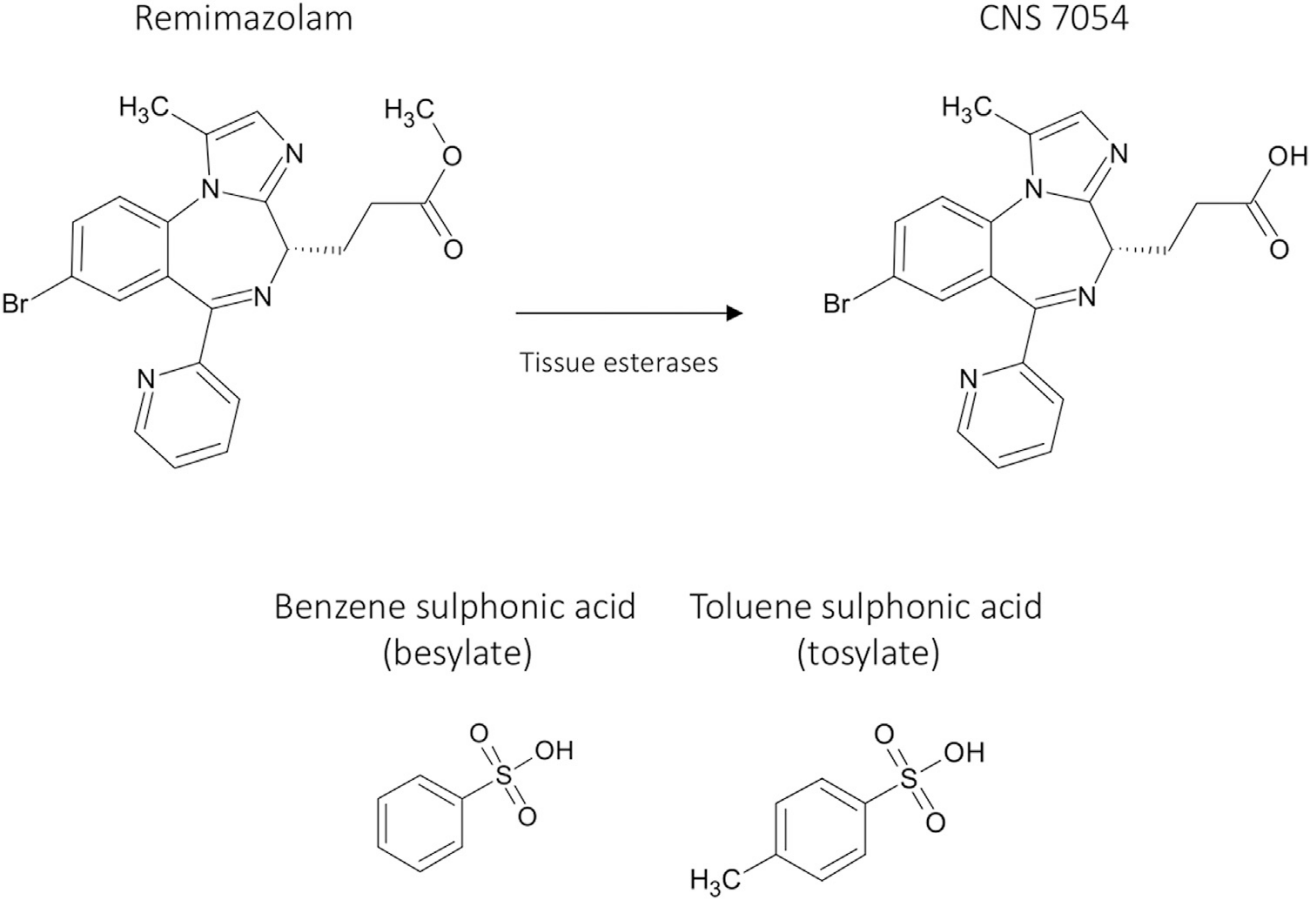
Remimazolam, salts of remimazolam, and the carboxylic acid metabolite, CNS 7054.

In the late 1990’s, the team at Glaxo Wellcome, who had discovered remifentanil (Feldman et al., 1991), embarked on another short-acting drug program focused on benzodiazepines. The project aimed to identify novel sedatives with a short and predictable duration of action (Pacofsky et al., 2002; Stafford et al., 2002; Feldman, 2020). GW502056 (remimazolam, Figure 2) was selected as a potential lead compound for this project based on parameters including rapid onset of sedation, short duration of action, wide separation of activity from the acid metabolite, and aqueous solubility. Some limited data on other potential lead compounds have been published. For example, CNS 7259 (Kilpatrick and Tilbrook, 2006), also termed compound 11a (Stafford et al., 2002)) has a high affinity for the human GABA_A_ receptor benzodiazepine site (Ki 6 nM), with the carboxylic acid metabolite being more than 400 times less active than the parent ester. Recovery from the loss of righting reflex in rodents was observed in under half the time of midazolam (25–30 mg/kg i.v.). In the micropig, CNS 7259 (0.05–1 mg/kg i.v.) induced sedation with a fast onset and a shorter offset than midazolam. CNS 7529 was not taken forward because of low aqueous solubility (Stafford et al., 2002).

Glaxo Wellcome halted the short-acting benzodiazepine program at the late lead optimization stage for strategic reasons, and the project was shelved for 2–3 years. Via the GSK Ventures initiative, the program, including all the compounds, patents, and associated data, was subsequently acquired in an “asset for equity transaction” (assignment in exchange for shares) by the UK-based private biotechnology company, TheraSci Ltd. TheraSci was itself simultaneously acquired by the UK-based public company, CeNeS PLC. Further pre-clinical characterization at CeNeS confirmed remimazolam as the lead candidate, and development studies to support clinical trials commenced. The first public information on the pharmacological and pharmacokinetic characterization of remimazolam (then CNS 7056) was presented at the American Society of Anaesthesiologists meeting in 2006. A series of a platform presentation and three posters were presented (Kilpatrick et al., 2006a; Kilpatrick et al., 2006b; Mutter et al., 2006; Tilbrook and Kilpatrick, 2006), followed a year later by a full paper published in Anesthesiology (Kilpatrick et al., 2007). The development program of remimazolam, using the besylate salt, was continued by CeNeS until it was acquired by the German-based company PAION AG in 2008. An investigational new drug application was filed with the U.S. Food and Drug Administration in 2008, and the first phase I study in healthy volunteers was completed later that year. Further development was undertaken by PAION and its licensees. The international non-proprietary name (INN) of remimazolam was approved in 2010 (International Nonproprietary Names for Pharmaceutical Substances (INN), 2010).

Using a slightly different (tosylate) salt form of remimazolam (Figure 2), a second development program was later initiated by Jiangsu Hengrui Pharmaceutical Co. Ltd. in China. The selection of the different salt was presumably a maneuver to circumvent the existing patent protection of the compound’s salts and polymorphic forms (Tilbrook and Bitt, 2006).

Remimazolam besylate has recently been approved for general anesthesia in Japan (Keam, 2020; Masui, 2020) and South Korea, procedural sedation in the United States (FDA, 2020b), China, and Europe (EMA, 2021). Compassionate use of remimazolam besylate for sedation in intensive care (ICU) has also been approved in Belgium (FAMHP, 2020). The tosylate salt has been approved for procedural sedation in China (Wang and Sun, 2020).

## Remimazolam

Alternative names for remimazolam include, GW502056, CNS 7056, CNS 7056B, CNS 7056BS, ONO-2745, and HR 7056. The chemical name of the base is methyl 3-[(4S)-8-bromo-1-methyl-6-pyridin-2-yl-4H-imidazo[1,2-a][1,4]benzodiazepin-4-yl] propanoate. Two salt forms have been developed: besylate and tosylate (Figure 2).

Remimazolam besylate is formulated as a lyophilized product for reconstitution (FDA, 2020a) but has sparing aqueous solubility, meaning that relatively large administration volumes need to be employed to accommodate the doses required in the anesthesia setting (Hirata et al., 2020).

### Pharmacology

Remimazolam shows high affinity (Ki∼30 nM) for the GABA_A_ receptor benzodiazepine site without significant activity at off-target receptors, ion channels, or enzyme sites that have been tested (Kilpatrick et al., 2007). Remimazolam weakly inhibits the hERG tail current with estimated EC_25_ and EC_50_ values of 62 and 207 μM (Kleiman et al., 2020). The compound showed positive modulation of the four major subtypes of the GABA_A_ receptor that respond to benzodiazepines (α_1_, α_2_, α_3_, α_5_) and in that respect is very similar to “classical” benzodiazepines such as midazolam. In these electrophysiology studies, remimazolam showed slightly higher potency at the α_1_ subtype of the GABA_A_ receptor (EC_50_ = 0.36 µM). The α_1_ subtype is the site associated with sedative, anterograde amnesic, and anticonvulsant effects of benzodiazepines (Rudolph et al., 1999; McKernan et al., 2000; Rudolph and Möhler, 2004).

The carboxylic acid metabolite of remimazolam, CNS 7054, was 300–400 times weaker (Ki∼10,000 nM) at the benzodiazepine site of the GABA_A_ receptor. CNS 7054 also showed no significant activity at off-target sites (Kilpatrick et al., 2007), and concentrations of up to 100 μM did not inhibit the hERG tail current (Kleiman et al., 2020).

Rodent *in vivo* studies showed that remimazolam induced dose-related deep sedation with fast onset and offset (Kilpatrick et al., 2007). The offset was quicker than observed for midazolam. Pre-treatment with the benzodiazepine receptor antagonist, flumazenil (Hunkeler et al., 1981) inhibited the sedative effect of remimazolam. This finding supports the suggestion that its sedative effects were caused by activating the benzodiazepine site of the GABA_A_ receptor. Inhibition of firing of neurons of the substantia nigra pars reticulata of the rat brain was also seen, with very rapid recovery after dosing ceased. This area of the brain receives prominent GABAergic innervation by striato-nigral afferents. The inhibition of firing observed provides further support for the GABA_A_ receptor being the site of action (Kilpatrick et al., 2007).

Upton and colleagues conducted an extensive analysis of remimazolam’s pharmacodynamics and pharmacokinetics in sheep (Upton et al., 2008, 2009; Upton et al., 2010). The pharmacokinetic studies are covered in *Pharmacokinetics*. The pharmacodynamic analysis revealed an apparent dose-related sedative effect of remimazolam (measured using EEG). There was a short duration of action without significant cardiovascular or respiratory depression (Upton et al., 2008; Upton et al., 2010). In a comparator study, remimazolam produced sedation with more rapid onset and offset rates than midazolam and propofol (Upton et al., 2009). The effects evoked by all three agents on the cardiovascular and respiratory systems were proportional to the sedation depth.

Only a relatively small number of studies of remimazolam pharmacology have been published in peer-reviewed journals in the decade since the early work described above. Liu and colleagues describe the sedative profile of remimazolam and some closely related derivatives in rats and rabbits (Liu et al., 2016). Bevans and colleagues (Bevans et al., 2017) reported that remimazolam could induce sedation in mice via the inhalation route and that remimazolam potentiated the effects of remifentanil when both drugs were administered by that route to rats. Io et al. (Io et al., 2021) observed tolerance to remimazolam over a 28 days dosing period in the pig. However, the tolerance observed (assessed by the requirement to increase the dose to maintain the same sedative effect) was less than that seen with midazolam. Information on the sedative effects of acute bolus doses and short infusions of remimazolam in the pig is also provided, revealing a fast onset and offset profile. Kops et al. (Kops et al., 2021) describe dose-related sedative effects of remimazolam in the cynomolgus monkey with rapid onset. They also report strong synergism between the effects of remimazolam and remifentanil. Watanabe et al. (Watanabe et al., 2021) examined the actions of remimazolam on the ryanodine1 receptor and the variant associated with malignant hyperthermia in response to volatile anesthetics. No effects associated with the ryanodine1 receptor were observed, and the authors conclude that remimazolam is unlikely to induce malignant hyperthermia.

Separate from the sedative effect of remimazolam, it has been reported (Xu et al., 2021) that derivatives of remimazolam induce apoptosis in glioma cells via down-regulation of the NF-κB pathway. The mechanism involved is not clear. It is also not clear whether remimazolam itself has these effects; no data appear to be presented relating to remimazolam itself, although it is implied by the title of this publication. Recently, it has also been reported that remimazolam alleviates neuropathic pain via regulating the bradykinin B_1_ receptor and autophagy (Xie et al., 2021). Studies to examine inhibition by flumazenil and the effects of other benzodiazepines, such as midazolam, would be useful in order to determine whether this effect is mediated via the benzodiazepine site on the GABA_A_ receptor or some other effect of remimazolam or its metabolite.

While there are few published papers on the non-clinical characterization of remimazolam, many studies are known to have been conducted to support the regulatory filings. Some of the data from these are available publicly. For example, summaries of very extensive pharmacology, safety pharmacology, pharmacodynamic, drug interaction, and toxicology studies of remimazolam besylate are available on the Food and Drug Administration (FDA) website and the Japanese Pharmaceutical and Medical Devices Agency (PMDA) website, albeit in partially redacted form (Sokolowski, 2019; Mundipharma Co. Ltd., 2020).

### Metabolism

Esterase metabolism breaks remimazolam down to its carboxylic acid metabolite, CNS 7054 (Figure 2), and methanol. Early studies showed that remimazolam (base; CNS 7056X) (Tilbrook and Kilpatrick, 2006) was very rapidly metabolized to the acid metabolite CNS 7054 by homogenates of liver from human, rat, mouse, and mini-pig. Rapid metabolism was also observed in tissues other than the liver, including mini-pig and rat kidney and rat lung. CNS 7056X was stable in plasma from human, pig, and dog for at least 60 min. The breakdown of remimazolam largely via tissue esterases differentiates it from many other short-acting ester drugs, where blood esterases usually play a predominant role (e.g., remifentanil (Feldman et al., 1991) and esmolol (Erhardt et al., 1982)).

**FIGURE 3:**
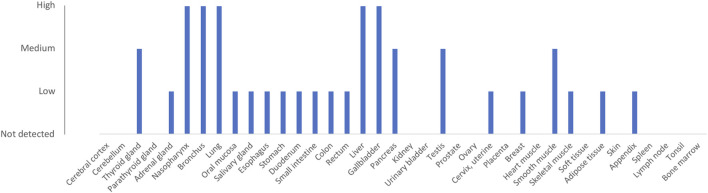
Protein expression of CES1 (ENSG00000198848). Data are derived from the Human Protein Atlas database (Uhlén et al., 2015; CES1 protein expression, 2021b). Protein was detected using immunocytochemistry with antibodies HPA012023 and HPA046717.

The major esterase responsible for remimazolam metabolism is often reported to be CES1 (Freyer et al., 2019; Sneyd and Rigby-Jones, 2020; Zhou et al., 2020a). However, no data appears to have been published to support this to date. Carboxylesterase 1 (CES1) is responsible for the metabolism of a number of drugs, including methylphenidate, clopidogrel, and the conversion of angiotensin-converting enzyme inhibitor prodrugs to the active drug (Laizure et al., 2013). It is highly expressed in the liver, gallbladder, and lung (Figure 3) (Uhlén et al., 2015; CES1 protein expression, 2021b). The genotype-tissue expression (GTEX) gene expression database (Carithers et al., 2015) (V8) shows that the liver is the organ with the highest expression, with a median number of CES1 transcripts per million (TPM) of 403 (from 226 individuals). The lung is the tissue showing the second-highest expression in the human body with 180 TPM. Other tissues with more than 50 TPM include the colon, major arteries, and adipose tissue (CES1 gene expression, 2021a). Transcript expression of CES1 in whole blood is relatively low (12 TPM). Overall, while expression of the protein and mRNA transcripts of CES1 is highest in the liver, the high expression in other tissues, including lung, suggests that extra-hepatic esterase metabolism of remimazolam (to CNS 7054) may be significant.

Significant variation in CES1 activity has been observed, and several genetic variants of CES1 associated with impaired enzyme activity have been identified (Wang et al., 2017; Gabriele et al., 2018). Individuals carrying these variants may be at risk of extended effects of remimazolam and other drugs metabolized by this enzyme. Extended effects of remimazolam have occasionally been observed (Zhou et al., 2020a; Yamamoto et al., 2021b), and further evaluation of the correlation of genetic variations in CES1 and the pharmacokinetics of remimazolam are warranted as well as interaction studies with other drugs known to be metabolized by this enzyme. Alcohol is a known inhibitor of CES1 (Parker et al., 2015) and is reported to increase the exposure (C_max_) to remimazolam by 1.2–2.1 fold in a dose-dependent manner (Pesic et al., 2020b).

In an additional analysis to their phase I study in Chinese subjects (Sheng et al., 2020), an evaluation of the effect of genetic single nucleotide polymorphisms in the vitamin D receptor, cytochrome P450 3A4, and 3A5, as well as cytochrome P450 oxidoreductase on the pharmacokinetics of remimazolam and CNS 7054 was conducted (Hu et al., 2021). A small effect of the rs4516035 and rs1544410 variants in the vitamin D receptor on the pharmacokinetics of CNS 7054 and remimazolam, respectively, was found. The authors note that this was a small study, and further data are required.

Zhou et al. (Zhou et al., 2017) evaluated the metabolite profile of remimazolam (tosylate) in plasma and urine after intravenous administration to man. 2 h after administration, the relative percentage area in mass spectrometry analysis of plasma was 0.33% remimazolam, 99.67% CNS 7054, and no other metabolites were detected. In urine, after 4 h, the percentages were 0.27% remimazolam and 98.63% CNS 7054. The remaining ∼1% consisted of two oxidation products and two glucuronidation products of CNS 7054. Sheng et al. (Sheng et al., 2020) report that after an acute dose of remimazolam, almost no parent compound was recovered in the urine (0–24 h), but the percentage of CNS 7054 recovered in the urine was between 70 and 90. These data support the hypothesis that the primary route of remimazolam metabolism is via esterases and that CYP enzymes do not play a significant role.

### Pharmacokinetics

Early studies in a pig model revealed that remimazolam (1.5 mg/kg/h for 15 min, i.v.) had a short half-life (18 min), a small volume of distribution (Vss 440 ml/kg), and very rapid clearance (35 ml/min/kg) (Mutter et al., 2006). Richard Upton’s group presented detailed pharmacokinetic data from dose-ranging (i.v.) pharmacokinetic/pharmacodynamic studies in the sheep in 2010 (Upton et al., 2010). High clearance, small distribution volumes, and a rapid onset and offset of sedation with predictable effects over a wide dose range were observed. The kinetics of arterial sampling of CNS 7056 was linear with dose and described by a three-compartment model (volumes: 1.9, 3.9, and 79 L) with clearance of 4.2 L/min and clearances between the compartment of 2.85 and 1.44 L/min. A mean residence time of 8.1 min was calculated for remimazolam. The t_1/2,Ke0_ (time for the concentration in the effect compartment to achieve 50% of the concentration in plasma) was 1.78 min.

The first human study of remimazolam (i.v.) included a detailed pharmacokinetic analysis from arterial and venous blood (Antonik et al., 2012). The pharmacokinetics of remimazolam were linear with dose, and its systemic clearance of 70.3 L/h was approximately three times that seen with midazolam. The steady-state volume of distribution was 88.1 L, terminal half-life 0.75 h, and mean residence time 0.51 h. Phase I single ascending dose trials of remimazolam besylate (Doi, 2014; Sheng et al., 2020) and remimazolam tosylate (Chen et al., 2020b) in Japanese and Chinese subjects report a similar profile to the initial phase I study with linear pharmacokinetics, high clearance, and short half-life. Pharmacokinetic analysis of remimazolam besylate administered by continuous infusions has been reported from volunteer studies (Sheng et al., 2020; Schüttler et al., 2020). The pharmacokinetic profiles were similar in both studies and consistent with those from single-dose studies.

The bioavailability of remimazolam after intranasal delivery of a powder formulation (10–40 mg) was ∼50% (Pesic et al., 2020a). Reduced bioavailability with increasing dose of solution formulation (47–26%) was reported by the same authors, but this is explained as likely to be due to swallowing of excess fluid when larger volumes were employed. The calculated elimination half-lives of remimazolam after intranasal administration (10–40 mg, powder or solution) ranged from 0.7–1.2 h, compared with 0.49 h after intravenous administration. When administered orally, the bioavailability of remimazolam is very low (1.1–2.2%) (Pesic et al., 2020a). Elimination half-lives could be calculated, however. These ranged from 0.33–0.69 h (60–480 mg) compared with 0.44 h after intravenous administration (1.25–2.58 mg).

Analysis of the pharmacokinetics of the acid metabolite, CNS 7054, has usually been conducted simultaneously with that of remimazolam. A profile of a small volume of distribution together with a longer mean residence time and slower clearance compared to remimazolam is routinely reported in man and large animals (Mutter et al., 2006; Upton et al., 2010; Antonik et al., 2012; Pesic et al., 2020b; Pesic et al., 2020a; Schüttler et al., 2020; Sheng et al., 2020; Chen et al., 2020b). For example, Antonik et al. (Antonik et al., 2012) report an apparent clearance of 4.22 L/h, a volume of distribution of 17.5 L, a half-life of 2.89 h, and a mean residence time of 3.6–5.1 h. The pharmacokinetics of CNS 7054 has been reported to fit best to a two-compartment model in sheep (Upton et al., 2010) and man (with a transit compartment to account for metabolite formation) (Schüttler et al., 2020).

### Pharmacokinetic/pharmacodynamic Modeling

A summary of PK/PD modeling analyses conducted with remimazolam, based on human data, is presented in Table 1. A physiologically based recirculation model was fitted to the pharmacokinetics of remimazolam from the initial clinical trial. In analyses since then, three-compartment models have been employed. Pharmacodynamic biomarker assessments have focused on EEG measures such as the bispectral index (BIS) score (Bower et al., 2000) and scores from the Modified Observer’s Assessment of Alertness/Sedation (MOAA/S) scale (Chernik et al., 1990). Some of the reasons to switch to a 3-compartment pharmacokinetic model are logical, e.g., reduced complexity and to facilitate dosing technologies such as target-controlled infusion (Schüttler et al., 2020; Sneyd and Rigby-Jones, 2020). Other reasons for dismissing the Wiltshire model are less logical, e.g., to claim it is “discredited” (Zhou et al., 2020a) because it assumes lung metabolism. This is on the basis of the ratio of liver/lung CES1 mRNA selected from a small study (Gabriele et al., 2018). Zhou et al., do not present any empirical data and contradictory data from large datasets on protein and transcript expression exist showing a high level of expression of the CES1 gene/protein in the lung and other tissues (see *Metabolism*).

**TABLE 1:** Population pharmacokinetic (PK) and pharmacodynamic (PD) models of remimazolam. MOAA/S = modified observer's assessment of alertness and sedation. BIS = bispectral index. EEG = electroencephalogram. NA = not available/applicable. *JapicCTI-111495 not included in this study. Note that data from some of the studies referenced by Trial ID have not yet been published in full.

Analysis	Trial ID	Salt form	PD measure	PK model	References
PK/PD	CNS 7056-001	Besylate	MOAA/S, BIS	Physiologically based recirculation	Wiltshire et al. (2012)
PK/PD	NCT01970072	Tosylate	MOAA/S, BIS	Three compartment	Zhou et al. (2018)
PK/PD	EudraCT 2017-000455-12	Besylate	MOAA/S	Three compartment	Schüttler et al. (2020)
PD	EudraCT 2017-000455-12	Besylate	EEG, MOAA/S	Three compartment	Eisenried et al. (2020)
PD	JapicCTI111495NCT01937767JapicCTI121973 NCT03661489	Besylate	Loss of consciousness, BIS, Narcotrend	NA	Lohmer et al. (2020)
PK/PD	CNS 7056-001 ONO-2745-01 ONO-2745–02 NCT01790607 EudraCT 2017-000455-12 CNS 7056–002 NCT01145222 NCT02290873 JapicCTI111495 NCT02296892 NCT02532647	Besylate	BIS	Three compartment	Zhou et al., (2020a)
PK		Besylate	NA	Three compartment	Zhou et al., (2020b)
PK/PD, Markov*		Besylate	MOAA/S	Three compartment	Zhou et al. (2021)

PK/PD modeling supports the observation of rapid onset and offset of effect. The t_1/2,Ke0_ parameter reflects the time required for the concentration in the effect compartment to reach 50% of the concentration in the plasma or the delay between cause and onset of effect. Calculated t_1/2,Ke0_ values for remimazolam are usually reported in the range of 1–5 min (Wiltshire et al., 2012; Zhou et al., 2020a; Eisenried et al., 2020; Schüttler et al., 2020; Zhou et al., 2021). In a study of remimazolam tosylate, longer t_1/2,Ke0_ values (=ln (2)/k_e0_) of 8.1 and 13.9 min were calculated for BIS monitoring and MOAA/S, respectively (Zhou et al., 2018). The reason for this difference from the other analyses is not clear.

A desirable feature in the anesthesia setting is that the elimination half time of a drug does not change significantly in relation to the duration of dosing. The context-sensitive half-time is a measure of this behavior, where the context is the duration of infusion. For remimazolam, the context-sensitive half-time appears to be largely insensitive to infusion duration, reaching a maximum after around 2 h at 6–11 min (Wiltshire et al., 2012; Eisenried et al., 2020; Schüttler et al., 2020). The predicted insensitivity of context-sensitive half-time with infusion duration calculated for remimazolam compares favorably to other hypnotics and opiates (Johnson, 2012).

Schüttler et al. (Schüttler et al., 2020) present calculated effect-site EC_50_s for MOAA/S score from 4 (mild sedation: lethargic response to name spoken in normal tone) to 0 (unconscious: no response to painful trapezius squeeze) in the range of 337–1,579 ng/ml. Remimazolam is 92% bound in plasma (Freyer et al., 2019). Data from brain homogenate binding does not appear to have been published, so corrections for bound/free compound cannot be made. The effect-site concentrations, uncorrected for bound drug, however, equate to concentrations of remimazolam of ∼0.77–3.6 µM, which compare with the observed EC_50_ of 0.36 µM at the recombinant human α_1_ GABA_A_ receptor subtype generated from *in vitro* electrophysiology studies and the Ki of 0.03 µM from radioligand binding assays (Kilpatrick et al., 2007).

Modeling to establish dosing schedules, with concomitant analgesic, in phase III procedural sedation and anesthesia trials and the marketed products has been conducted (Zhou et al., 2020a; Zhou et al., 2020b; Lohmer et al., 2020; Zhou et al., 2021). Dosing without correction for body weight is supported in procedural sedation (Wiltshire et al., 2012; Zhou et al., 2020b; Schüttler et al., 2020; Zhou et al., 2021). While some differences in the behavior of remimazolam are observed related to age, race, gender, obesity status, weight, and ASA class, covariate analysis indicates that major dose adjustments are not needed for the majority of patients (Zhou et al., 2018, 2021; Zhou et al., 2020a; Zhou et al., 2020b; Lohmer et al., 2020).

### Clinical Data

Multiple clinical trials of remimazolam have been conducted and published. They can be broadly divided into examining the effects of remimazolam in 1) human volunteers, 2) patients undergoing procedures under sedation, and 3) patients undergoing general anesthesia. These are summarized in Tables 2 – 4. Since remimazolam has reached the market, case reports of its clinical use are also starting to be published. These are summarized in Table 5. Several further remimazolam trials are registered on clinical trial databases; of note are studies examining its use for sedation in intensive care. However, the results of these are yet to be published.

**TABLE 2:** Published studies of remimazolam trials in human volunteers. NA = not available/applicable, PK = pharmacokinetics, PD = pharmacodynamics, iv = intravenous, ECG = electrocardiogram. N = number of subjects. *Note that the doses appear to represent the molecular weight of the salt and not to been corrected to base in these studies (Zhou et al., 2018).

Trial ID	N	Endpoints	Salt	Dose	Route	Infusion duration	Co-treatment	Comparator	Ref
CNS 7056-001	81	Safety, PK, PD	Besylate	0.01–0.3 mg/kg	iv	1 min	NA	Placebo, midazolam	Antonik et al. (2012); Wiltshire et al. (2012)
CNS 7056-002 (A)	6	Flumazenil reversal	Besylate	0.25 mg/kg	iv	1 min	Flumazenil	Placebo	Worthington et al. (2013)
CNS 7056-002 (B)	45	Colonoscopy: Safety, efficacy	Besylate	0.04–0.1 mg/kg + top-ups	iv	1 min	Fentanyl	NA	Worthington et al. (2013)
ONO-2745-01	42	Safety, PK, PD	Besylate	0.05–0.5 mg/kg	iv	1 min	NA	NA	Doi, (2014)
ONO-2745-02	10	Safety, PK, PD	Besylate	1 mg/kg/h	iv	Up to 60 min	NA	NA	Doi, (2014)
ChiCTR 1800015185	62	Safety, PK, PD	Besylate	0.025–0.4 mg/kg	iv	1 min	NA	Placebo, midazolam	Sheng et al., (2020)
ChiCTR 1800015186	12	Safety, PK, PD	Besylate	0.2 mg/kg then 1 mg/kg/h	iv	1 min, 2 h	NA	Midazolam	Sheng et al., (2020)
NCT01970072	79	Safety, PK, PD	Tosylate	0.01–0.45 mg/kg*	iv	1 min	NA	Placebo, midazolam	Chen et al., (2020b)
NCT03444480	8	Flumazenil reversal	Tosylate	0.4 mg/kg then 1.5 mg/kg/h*	iv	1 min, 2 h	Flumazenil	NA	Chen et al., (2020b)
EudraCT 2017-000455-12	20	PK, PD	Besylate	5 mg/min, 3 mg/min, 1 mg/min	iv	5, 15, 15 min	NA	NA	Schüttler et al. (2020)
NCT03329014	12	Safety, PK, PD	Besylate	10–40 mg	Nasal	NA	NA	Placebo, iv remimazolam	Pesic et al., (2020a)
NCT04113564	14	Safety, PK, PD	Besylate	0.14 mg/kg	Oral	NA	NA	iv remimazolam	Pesic et al. (2020b)
NCT04113343 Part1	21	Safety, PK, PD	Besylate	60–480 mg	Oral	NA	NA	NA	Pesic et al. (2020b)
NCT04113343 Part2	11	Safety, PK, PD	Besylate	360 mg	Oral	NA	Alcohol	NA	Pesic et al., (2020b)
NCT04110535	40	Abuse potential	Besylate	5–10 mg	iv	1 min	NA	Placebo, midazolam	Schippers et al. (2020)
CNS 7056-005	57	ECG	Besylate	10–20 mg	iv	1 min	NA	Midazolam, moxifloxacin, placebo	Kleiman et al. (2020)
EudraCT 2017-000455-12	20	ECG	Besylate	5 mg/min, 3 mg/min, 1 mg/min	iv	5, 15, 15 min	NA	Placebo	Kleiman et al. (2020)

**TABLE 3:** Published clinical trials of remimazolam in procedural sedation. NA = not available/applicable, GI = gastrointestinal. N = number of patients.

Trial ID	Procedure	Phase	ASA class	N	Primary endpoint	Salt form	Dose	Co-treatment	Comparator	Ref
NCT00869440	Upper GI endoscopy	II	I - II	100	Success of procedure	Besylate	0.1–0.2 mg/kg	NA	Midazolam	Borkett et al. (2015)
NCT01145222	Colonoscopy	II	I - III	162	Success of procedure	Besylate	5–8 mg plus 2–3 mg top-ups	Fentanyl	Midazolam	Pambianco et al. (2016)
NCT02290873	Colonoscopy	III	I - III	461	Success of procedure	Besylate	5 mg plus 2.5 mg top-ups	Fentanyl	Midazolam, placebo	Rex et al. (2018)
NCT02296892	Bronchoscopy	III	I - III	446	Success of procedure	Besylate	5 mg plus 2.5 mg top-ups	Fentanyl	Midazolam, placebo	Pastis et al. (2019)
NCT02532647	Colonoscopy	III	III-IV	79	Success of procedure	Besylate	2.5–5.0 mg plus 1.25–2.5 mg top-ups	Fentanyl	Midazolam, placebo	Rex et al. (2021)
NCT03779061	Colonoscopy	III	I - II	384	Successful sedation	Tosylate	5 mg plus 2.5 mg top-ups	Fentanyl	Propofol	Chen et al., (2020a)
NCT03425474	Upper GI endoscopy	III	I - II	384	Successful sedation	Tosylate	5 mg plus 2.5 mg top-ups	Fentanyl	Propofol	Chen et al. (2021)
ChiCTR-2000038252	Hysteroscopy	II	I-II	82	Adverse events	Besylate	0.2 mg/kg/min then 1 mg/kg/h	Remifentanil	Propofol	Zhang et al. (2021)

**TABLE 4:** Published clinical trials of remimazolam in general anesthesia. NA = not available/applicable. N = number of patients.

Trial ID	Procedure	Phase	ASA class	N	Primary endpoint	Salt	Dose	Co-treatment	Comparator	Ref
JapicCTI111495	Various surgeries	II	I-II	85	Successful anesthesia	Besylate	Induction:Various maintenance 1 mg/kg/hr	Remifentanil, rocuronium	NA	Doi (2014); Doi et al. (2015)
NCT01937767	Cardiac surgery	II	NA	90	Successful anesthesia	Besylate	Induction 6–12 mg/kg/hr; maintenance ∼1 mg/kg/hr	Fentanyl/remifentanil, rocuronium	Propofol, sevoflurane	Probst et al. (2014, 2015); Bevilacqua et al. (2015)
JapicCTI121973	Various surgeries	II/III	I - II	375	Successful anesthesia	Besylate	Induction 6–12 mg/kg/hr; maintenance ∼1 mg/kg/hr (titration)	Remifentanil, rocuronium	Propofol	Doi et al., (2020a)
NCT03661489	Various surgeries	III	III	67	Successful anesthesia	Besylate	Induction 6–12 mg/kg/hr; maintenance 2 mg/kg/hr	Remifentanil, muscle relaxant	NA	Doi et al., (2020b)

**TABLE 5:** Case reports on the use of remimazolam besylate in clinical practice. NA = Not available/applicable, ECRP = Endoscopic retrograde cholangiopancreatography, MEP = Motor evoked potentials, EEG = electroencephalograph.

Procedure	Dose	Salt	Co-treatment	Comment	Ref
Spine surgery	Induction: 6 or 12 mg/kg/h; maintenance 0.5–1.5 mg/kg/h	Besylate	Remifentanil, rocuronium	MEP monitoring by EEG. Successful	Kondo et al. (2020)
Craniotomy	Induction 12 mg/kg/h; maintenance 1 mg/kg/h	Besylate	Remifentanil, fentanyl	Awake when infusion stopped. Successful	Sato et al. (2020)
ECRP	Induction 12 mg/kg/h; maintenance 1 mg/kg/h	Besylate	Remifentanil, rocuronium	High risk: Myotonic dystrophy. Successful	Fukuda et al. (2021)
Cardiac surgery	Induction 6 mg/kg/h; maintenance 0.6–1 mg/kg/h	Besylate	Remifentanil, fentanyl, rocuronium	Cardiopulmonary bypass. Successful	Saito et al. (2021)
Laminoplasty for spinal stenosis	Induction 12 mg/kg/h; maintenance 1 mg/kg/h	Besylate	Rocuronium	Re-sleeping observed after flumazenil reversal	Yamamoto et al. (2021b)
Craniotomy	Induction 6 mg/kg/h; maintenance 0.75–1 mg/kg/h	Besylate	Remifentanil flumazenil	Awakening during surgery with flumazenil. Successful	Yoshida et al., (2021b)
Craniotomy	NA	Besylate	NA	Concerns around pharyngeal reflex, blood pressure, and agitation on flumazenil reversal	Tachibana, Hayamizu and Yamakage, (2021)
Surgery: 1) stent removal and 2) tumor resection	Initially 10 and 13 mg bolus respectively plus additional doses	Besylate	Remifentanil/fentanyl, rocuronium	Reduced effect in long-term benzodiazepine users	Yoshikawa et al. (2021)
Cataract surgery, rigid laryngoscopy, bronchoscopy, intramedullary nailing, and hemiarthroplasty/hip fracture	5–20 mg	Besylate	NA	Reduced impact on neuropsychiatric function compared to existing sedative agents	Henson and Thompson, (2021)
Thyroid surgery	Induction 12 mg/kg/h; maintenance 1 mg/kg/h	Besylate	Remifentanil, rocuronium	Comparable anesthesia to existing drugs for neuromonitoring during thyroid surgery	Hayamizu et al. (2021)
Hand surgery	Induction 6 mg/kg/h	Besylate	Remifentanil, fentanyl, rocuronium	Anaphylaxis to remimazolam	Tsurumi et al. (2021)
MitraClip implantation in a patient with advanced heart failure	Induction 6 mg/kg/h; maintenance 0.15 mg/kg/h	Besylate	Remifentanil, rocuronium	Appropriate anesthetic management in a patient with severe cardiovascular disease	Satoh et al. (2021)
Cochlear implant in a patient with mitochondrial encephalomyopathy	Induction 0.2 mg/kg (1 min); maintenance 1 mg/kg/h	Besylate	Remifentanil	Successful use for general anesthesia in a patient with mitochondrial myopathy	Suzuki, Doi and Nakajima, (2021)

#### Volunteers

The data from the first clinical trial of remimazolam in volunteers was reported in two papers published in 2012, one describing the safety, pharmacokinetics, and pharmacodynamics (Antonik et al., 2012) and the other, a population pharmacokinetic and pharmacodynamic modeling analysis (Wiltshire et al., 2012). The study evaluated nine single, ascending doses of remimazolam besylate (0.01–0.3 mg/kg) and included placebo and midazolam control groups. Doses were selected on the basis of the pre-clinical large animal data and modeling. As observed using BIS or MOAA/S score biomarkers, dose-related sedation with rapid onset and offset was seen in the remimazolam groups. The pharmacodynamic profile from large animals translated very well to humans, with the lowest dose that induced sedative effects being 0.075 mg/kg as was predicted by modeling (Antonik et al., 2012). The drug was well tolerated, with no supplemental oxygen or ventilation requirement. Times to recovery were shorter in the remimazolam treated groups than the midazolam treated group. Very similar single ascending dose studies of remimazolam besylate have been conducted in Japanese (Doi, 2014) and Chinese populations, one with remimazolam besylate (Sheng et al., 2020) and one with remimazolam tosylate (Chen et al., 2020b). These three studies had very similar outcomes of a good tolerability profile and dose-related sedation with rapid onset and offset. The study in Japanese subjects (Doi, 2014) included an elderly group (>65 years), and no change in the profile associated with age was reported.

Infusion regimens of remimazolam have been evaluated in four volunteer studies, three using the besylate salt (Doi, 2014; Sheng et al., 2020; Schüttler et al., 2020) and one the tosylate (Chen et al., 2020b). In one study, a single dose of 1 mg/kg/h for up to 60 min was used (Doi, 2014). The other three employed an initial higher dose (0.2 mg/kg/min [1 min] (Sheng et al., 2020), 0.4 mg/kg [bolus] (Chen et al., 2020b), 5 mg/min [5 min] (Schüttler et al., 2020)) to induce sedation followed by a continuous infusion at a lower dose (1–2 mg/kg/h [2 h] (Sheng et al., 2020), 1.5 mg/kg/h [2 h] (Chen et al., 2020b) and 3 mg/min [15 min] followed by 1 mg/min [15 min] (Schüttler et al., 2020). Sedation was monitored by BIS or EEG monitoring and MOAA/S scoring biomarkers. These studies show a consistent theme of rapid onset of sedation, maintenance over the continuous infusion, and rapid recovery after the infusion was stopped.

Reversal of the sedation induced by remimazolam by flumazenil in a human study was first reported by Worthington et al., 2013 (Worthington et al., 2013), an observation that has since been confirmed in several studies (see *Flumazenil reversal*).

Further volunteer studies have been conducted to evaluate remimazolam’s cardiac electrophysiology effects, its bioavailability by other administration routes, and its abuse potential (Kleiman et al., 2020; Pesic et al., 2020a; Pesic et al., 2020b). The latter two points are addressed in *Adverse reactions and liabilities* and *Alternative indications and routes of administration*. The authors of the cardiac electrophysiology studies conclude that remimazolam does not prolong cardiac repolarization although they observe that an increase in QT_c_ interval may occur after bolus administration resulting from an increase in heart rate (Kleiman et al., 2020).

The pharmacodynamic effects of remimazolam can be readily measured in volunteers, but data correlating these effects with target engagement at the GABA_A_ benzodiazepine receptor are not available. Positron emission tomography technologies to measure *in vivo* binding to the benzodiazepine site of the GABA_A_ receptor are well established, e.g., using [^11^C] or [^18^F] derivatives of flumazenil (Persson et al., 1985; Gründer et al., 2001). It would be of interest to undertake studies to evaluate the benzodiazepine receptor engagement by remimazolam using this technique. The availability of such data may better inform modeling of the sedative actions of remimazolam.

#### Procedural Sedation

The sedation of patients to reduce pain, reduce anxiety and provide amnesia facilitates uncomfortable diagnostic procedures such as endoscopy (Dossa et al., 2021) and interventional procedures such as those employed in cardiology (Makkad, 2013). Several clinical trials of remimazolam as a sedative agent for procedural sedation have been conducted. The bulk have been in colonoscopy, upper gastrointestinal endoscopy, and bronchoscopy, and there is a single report of a trial in hysteroscopy (Table 3).

The first report of use in colonoscopy was a phase Ib dose-range finding remimazolam besylate study in volunteers (Worthington et al., 2013). Multiple doses were employed in a design utilizing initial (0.04–0.1 mg/kg) and top-up doses to maintain sedation for 30 min. Subjects also received a single dose of fentanyl. Successful sedation (judged by achieving a MOAA/S score of ≤4 on three repeat measurements, completion of the procedure, no requirement for rescue sedative medication, and no requirement for assisted ventilation) was achieved in >70% of subjects. Fast onset and offset of sedation were observed.

Three phase II studies of remimazolam besylate in procedural sedation have been published. The first was in 100 patients undergoing upper gastrointestinal endoscopy (Borkett et al., 2015), the second in 162 patients undergoing colonoscopy (Pambianco et al., 2016), and the third in 82 patients undergoing hysteroscopy (Zhang et al., 2021). A midazolam-treated group was included as a comparator in the first two studies. In the third study, propofol was the comparator drug. In the upper G.I. endoscopy study, no analgesic co-treatment was reported to be employed. Relatively modest success rates were achieved (32%, 56%, and 64% for 0.10, 0.15 and 0.20 mg/kg remimazolam, respectively) compared with 44% of patients for midazolam (0.075 mg/kg). In the colonoscopy study, the analgesic fentanyl was co-administered, and higher success rates were observed (>92% for the remimazolam groups and 75% for the midazolam group). The hysteroscopy study's success rates were 100% for both the remimazolam and propofol groups, with a faster recovery seen in the remimazolam group. The remimazolam group’s adverse event profile was superior to the propofol group in terms of hemodynamic fluctuation, excessive sedation depth, and low SpO2. There was a much lower incidence of injection pain in the remimazolam group.

Five phase III studies have been reported, three for remimazolam besylate and two for remimazolam tosylate. The doses routinely employed in the phase III studies were uncorrected for body weight and the same for both salts, i.e., an initial dose of 5 mg with additional top-ups of 2.5 mg as required.

The besylate studies are in colonoscopy (Rex et al., 2018, Rex et al., 2021) and bronchoscopy (Pastis et al., 2019) and include a colonoscopy study in a high-risk group (ASA class III-IV) (Rex et al., 2021). All three besylate studies were placebo-controlled with a midazolam comparator group (open label) and included co-treatment with the analgesic fentanyl. Success was defined as completion of colonoscopy, no need for an alternative sedative, or more than five top-ups in a 15 min period. Each trial met the primary endpoint, and the conclusion was reached for all studies that remimazolam was safe and effective for sedation during the procedures with rapid onset of effect and recovery. In these studies, the success rates in the remimazolam groups (80.6–91.3%) were considerably higher than the midazolam (13.3–32.9%) or placebo groups (0–4.8%). In all studies, the procedure was started sooner in the remimazolam treatment arm (5.1–8.0 min) than in the midazolam (13.3–32.9 min) or placebo control arms (12–21.9 min). The time to return to full alertness after the procedure was shorter in the remimazolam treatment arm (3.0–7.3 min) than in the midazolam (7.0–15.8 min) or placebo control arms (5.3–21.0 min). Hypotension was observed less frequently in the remimazolam arm in the larger colonoscopy study (Rex et al., 2018); otherwise, the safety profiles of the remimazolam and midazolam arms of these studies were comparable. The mean total dose of remimazolam used to complete the procedure in these studies ranged from 9.0–11.5 mg.

The tosylate phase III studies were conducted in colonoscopy (Chen et al., 2020a) and upper gastrointestinal endoscopy (Chen et al., 2021). Propofol was used as the comparator in these studies with a non-inferiority design. The primary endpoints were met in both studies with high sedation success rates for remimazolam that were statistically non-inferior (colonoscopy = 96.9%, upper gastrointestinal endoscopy = 97.3%) but numerically lower than those for propofol (100% in both studies). A slightly slower onset time was observed in the colonoscopy study after remimazolam treatment (100 s) than propofol treatment (75 s) with no difference in offset time. In the upper gastrointestinal study, patients treated with remimazolam also showed a slower onset time than propofol but had a faster offset time (time to fully alert, remimazolam 5.75 min, propofol 6.71 min). Hypotension was observed in fewer patients in the remimazolam arms of these studies (colonoscopy 23.7%; upper G.I. endoscopy 13.0%) than the propofol arms (colonoscopy 51.1%; upper G.I. endoscopy 42.9%). Respiratory depression was also significantly lower in the remimazolam arms of these studies (colonoscopy 3.1%; upper G.I. endoscopy 1.1%) than the propofol arms (colonoscopy 16.9%; upper G.I. endoscopy 6.9%). Mild gait disorders and dizziness were reported with a similar incidence in the two arms of the colonoscopy study. Injection site pain was only observed for one subject in the remimazolam arms in the two studies combined compared with 10–16% of the propofol treatment arms.

#### General Anesthesia

Benzodiazepines have been employed to provide anesthesia’s hypnotic component, but they are not widely used because of the variability of effect, extended duration of action, and lack of control of anesthesia depth (Gamble et al., 1981; Saari et al., 2011). With the observation of the controllable short-acting effects of remimazolam, it became clear that it may be a useful agent in anesthesia (for comment see (Masui, 2020; Sneyd and Rigby-Jones, 2020)).

Reports of four clinical trials of the use of remimazolam for total intravenous anesthesia (TIVA) have been published to date (Table 4), two as full papers reporting a phase IIb/III trial and a phase III trial (Doi et al., 2020a; Doi et al., 2020b), and two reporting phase II trials, one as a short report and abstract (Doi, 2014; Doi et al., 2015) and one as a series of abstracts (Probst et al., 2014, 2015; Bevilacqua et al., 2015). All are trials with remimazolam besylate and employ continuous infusions of remimazolam, co-administered with an opiate analgesic (fentanyl or remifentanil) and neuromuscular blockade, usually with rocuronium. In contrast to procedural sedation, doses corrected for body weight were employed for this indication. Data from the phase II studies are relatively sparse, but successful anesthesia, as defined by no requirement for rescue medication, is reported in both trials after induction and maintenance infusion of remimazolam (Doi, 2014; Probst et al., 2014). Rapid onset of effect was observed as well as a rapid recovery after the surgery. There were no signs of awakening during the surgery nor any memory of the procedure (Doi et al., 2015). A reduced requirement for vasopressor treatment compared with propofol/sevoflurane was observed in the cardiac surgery study (Probst et al., 2015).

Significantly more detailed information is available for the phase II/III and III studies. For induction of anesthesia, a short infusion of 6 or 12 mg/kg/h was used, and for maintenance of anesthesia, a dose of 1–2 mg/kg/h. The primary endpoints were successful anesthesia as defined by the lack of intra-operative awakening or recall, no requirement for rescue sedation, and no body movement. The primary endpoints were met with successful anesthesia achieved for all subjects in both studies. Time to loss of consciousness was slightly longer for remimazolam (102 s and 88.7 s for 6 mg/kg/h and 12 mg/kg/h induction doses, respectively) when compared to propofol (78.7 s) (Doi, et al., 2020a). Time from ceasing the infusion of the drug to extubation was also slightly longer in the remimazolam arms (19.2 min) compared to propofol (13.1 min) (Doi, et al., 2020a). There was a lower incidence of hypotension in subjects receiving remimazolam (22%) than those receiving propofol (49.3%). Fewer patients treated with remimazolam required vasopressors (41.3%) or treatment for bradycardia (6.3%) compared with the propofol arm (64.0 and 9.3%, respectively). Pain on injection was reported for 18.7% of subjects treated with propofol but none treated with remimazolam. The only adverse drug reaction that was higher in the remimazolam arms vs. propofol arms was nausea and vomiting (7 and 6% respectively for remimazolam and 5.3 and 4% for propofol). One of the studies examined clinically vulnerable patients (ASA Class III). Efficacy and safety data were similar to those in ASA I and II patients (Doi, et al., 2020b).

The mean length of procedure in the large phase II/III study was ∼150 min, and the mean total dose of remimazolam was 3.47 mg/kg (Doi et al., 2020a). This translates to ∼240 mg for a 70 kg individual, compared with ∼10 mg for the procedural sedation studies (*Procedural sedation*).

#### Case Reports

Since reaching the market, several case reports have been published on the use of remimazolam (Table 5). The use in awake craniotomy is of particular interest, especially incorporating the planned use of flumazenil to reverse sedation during the procedure (Yoshida et al., 2021). However, Tachibana et al. (Tachibana et al., 2021) have questioned the effectiveness of remimazolam for craniotomy based on observation from two subjects. They make three points. Firstly, that increased sputum is produced compared to propofol due to the lower suppression of the pharyngeal reflex by remimazolam; secondly, that, because remimazolam has a lower effect on blood pressure compared to propofol, surgical difficulties can result due to higher intracranial pressure and thirdly that agitation was observed in one subject upon flumazenil reversal. Clearly, further study is required on the use of remimazolam in craniotomy to understand better the risk/benefit balance.

Successful use of remimazolam has been described in spine surgery incorporating motor evoked potential monitoring (Kondo et al., 2020), thyroid surgery with neuromonitoring (Hayamizu et al., 2021), and cardiac surgery with cardiopulmonary bypass (Saito et al., 2021). Remimazolam’s successful use in high-risk patients, including endoscopic retrograde cholangiopancreatography in a patient with impaired respiratory function due to myotonic dystrophy (Fukuda et al., 2021), MitraClip implantation in a patient with advanced heart failure (Satoh et al., 2021), and cochlear implant in a patient with mitochondrial myopathy (Suzuki et al., 2021) has been published.

In the procedural sedation setting, Henson and Thompson (Henson and Thompson, 2021) report that sedation with remimazolam resulted in a lower “insult” to neuropsychiatric function in geriatric patients undergoing a variety of procedures compared to existing agents.

There has been an observation of re-sedation after flumazenil reversal (Yamamoto et al., 2021b; Yamamoto et al., 2021a). This may have been due to residual remimazolam, the metabolite CNS 7054 building up to levels that can activate the GABA_A_ receptor or a combination of both, potentially as a result of compromised organ function. There are also reports of precipitation of remimazolam in Ringer’s solution (Yoshida et al., 2021; Matsuo et al., 2021; Sasaki et al., 2021). A single report of anaphylaxis resulting from the administration of remimazolam has been published (Tsurumi et al., 2021).

Further case reports that shed light on the utility of remimazolam in practice can be expected in the coming years.

### Adverse Reactions and Liabilities

The two salts of remimazolam have very similar adverse reaction profiles, which are in line with those observed with other classical benzodiazepines. The most frequently observed adverse reactions in the procedural sedation setting are changes in blood pressure and heart rate, reduced respiratory rate, and vomiting. The incidence of these events was comparable with midazolam comparator groups (Rex et al., 2018; Pastis et al., 2019). In high-risk patients (ASA class III/IV), a safety profile similar to that in low-risk patients was observed (Rex et al., 2021). Compared with propofol in procedural sedation, remimazolam showed a lower incidence of hypotension, respiratory depression, and injection site pain (Chen et al., 2020a; Chen et al., 2021; Zhang et al., 2021).

The most common adverse reactions in anesthesia are hypotension, vomiting, and nausea, (Doi, et al., 2020b; Doi, et al., 2020a). The safety profile of remimazolam in high-risk patients (ASA class III) was in line with corresponding results from ASA class I and II patients (Doi, et al., 2020b). In comparison to propofol, the incidence of hypotension, requirement to treat for bradycardia, and injection site pain were lower in the remimazolam arms, while propofol may be slightly superior with respect to postoperative nausea and vomiting (PONV) (Doi, et al., 2020a). There was also a trend toward reduced nausea and vomiting in the propofol arm of the phase III colonoscopy study compared with remimazolam tosylate (Chen et al., 2020a). Both propofol and midazolam are recognized to have anti-emetic properties (Soppitt et al., 2000; Ahn et al., 2016). Further studies to evaluate the incidence of PONV after remimazolam treatment, in comparison to propofol, would be of interest.

Benzodiazepines are known to induce tolerance and dependence on repeated use and to have abuse potential (O’brien, 2005). Remimazolam is likely to have the same liabilities, but they are probably mitigated to an extent because of the short duration of effect and the requirement for intravenous administration. Indeed, Io et al. (Io et al., 2021) observed tolerance to the effects of remimazolam over a 28 days dosing period in the pig, but note that the tolerance observed (assessed by the requirement to increase the dose to maintain the same sedative effect) was less than that seen with midazolam. As might be predicted, a reduced sedative effect of remimazolam has been observed in subjects with an existing tolerance to benzodiazepines resulting from long-term use equivalent to 15–20 mg diazepam per day (Yoshikawa et al., 2021).

In a comparative study of recreational drug users, it was concluded that remimazolam does have the potential for abuse but that this was comparable to or lower than that of midazolam (Schippers et al., 2020). The authors note midazolam itself has a low potential for intravenous abuse. Pesic et al. (Pesic et al., 2020a) conducted a trial in female subjects and report that remimazolam besylate does not have the potential for misuse to facilitate sexual assaults because of its very low oral bioavailability and bitter taste.

Synergistic pharmacological interaction between benzodiazepines and opioid analgesics is well established. This is usually beneficial in both procedural sedation and anesthesia settings. The synergy with opioid analgesics has been clearly established for remimazolam (Bevans et al., 2017; Zhou et al., 2020a; Kops et al., 2021; Zhou et al., 2021).

Remimazolam is thought to have a lower potential for pharmacokinetic drug interactions than midazolam. Midazolam is metabolized primarily by cytochrome P450 3A4, a common drug metabolism enzyme that is inhibited by several drugs leading to interaction issues (Yuan et al., 1999). One factor to consider, however, is that the reported major metabolizing enzyme of remimazolam, CES1, is inhibited by naturally occurring agents such as flavonoids and fatty acids (Crow et al., 2010; Wang et al., 2018). Alcohol, an inhibitor of CES1, has been shown to increase exposure to remimazolam (Pesic et al., 2020a).

### Alternative Indications and Routes of Administration

The properties of remimazolam potentially make it a useful agent for ICU sedation, especially to facilitate light sedation protocols and rapid awakening (Barr et al., 2013). The original Japanese licensee for remimazolam besylate, Ono Pharmaceutical Co., conducted a study in ICU sedation. No peer-reviewed paper report of this study appears to be available; however, Zhou et al. (Zhou et al., 2020a) note that “data from 49 Japanese patients in the intensive care unit (ICU) showed that seven subjects treated longer than 24 h had higher than expected concentrations of remimazolam for samples collected >24 h after the initiation of remimazolam dosing.” Further evaluation of the extended effects observed is warranted, as is a further study of the potential utility of remimazolam in ICU sedation. Note that remimazolam has already been approved in Belgium for compassionate use in ICU sedation (FAMHP, 2020), owing to a shortage of current medications resulting from the COVID-19 crisis.

Remimazolam is unlikely to find use by the oral route because of its very low bioavailability (∼1–2%), which is largely a result of extensive first-pass metabolism (Pesic et al., 2020a). Bioavailability by the intranasal route is significantly higher (∼50%), and sedative effects were observed after administration to human volunteers via this route. The formulations that were employed in this study (powder and the intravenous formulation of remimazolam besylate), however, caused significant nasal discomfort/pain (Pesic et al., 2020b). Otherwise, the drug was reported to be safe and well-tolerated by this route. If a more tolerable formulation can be developed, intranasal delivery for settings where intravenous administration is problematic such as in epilepsy, agitation or for pediatric sedative use (Fantacci et al., 2018; Shioji et al., 2021), may be feasible. Inhalation has also been proposed as an alternative delivery route, and some animal data supports this, showing that inhaled remimazolam potentiates the effects of inhaled remifentanil (Bevans et al., 2017).

### Flumazenil Reversal

Flumazenil is an antagonist to the positive allosteric modulator effects of benzodiazepines at the GABA_A_ receptor (Hunkeler et al., 1981). The compound is approved for clinical use for reversal of benzodiazepine effects, usually resulting from overdose. Reversal or prevention of the effects of remimazolam by flumazenil has been clearly demonstrated in pre-clinical (Kilpatrick et al., 2007) and clinical studies (Worthington et al., 2013; Chen et al., 2020b). In the anesthesia phase III studies, flumazenil was used as a reversal agent in subjects (∼5–10%) who had not awoken within 30min of the termination of remimazolam infusion (Doi, et al., 2020b; Doi, et al., 2020a). The availability of a reversal agent provides a safety feature in case of overdose or adverse events that is not available for many other anesthetics, including propofol (Sneyd and Rigby-Jones, 2020). An important factor to consider, however, is that flumazenil is itself an ester-based drug and has a short half-life and duration of action (Lauven et al., 1985), so multiple doses may be required to avoid re-sedation events.

The planned use of flumazenil in combination with remimazolam to control sedation levels precisely may open new avenues in procedural sedation and anesthesia. The use of flumazenil to reverse remimazolam-induced anesthesia during a craniotomy procedure to awaken the patient is an interesting example (Yoshida et al., 2021).

## Conclusion and Future Directions

The aim of this review is to provide an analysis of, and perspective on, published non-clinical and clinical information on remimazolam. The recent introduction of this compound to the market appears to provide a valuable addition to the armory of agents available for procedural sedation and anesthesia. Remimazolam has the pharmacological profile of a classical benzodiazepine but is differentiated by its ester group and rapid metabolism, via tissue esterases, to a significantly less active metabolite. The compound exhibits rapid pharmacokinetics and pharmacodynamics with relatively small effects of covariates such as age, gender, race, obesity status, ASA class, and weight.

**FIGURE 4:**
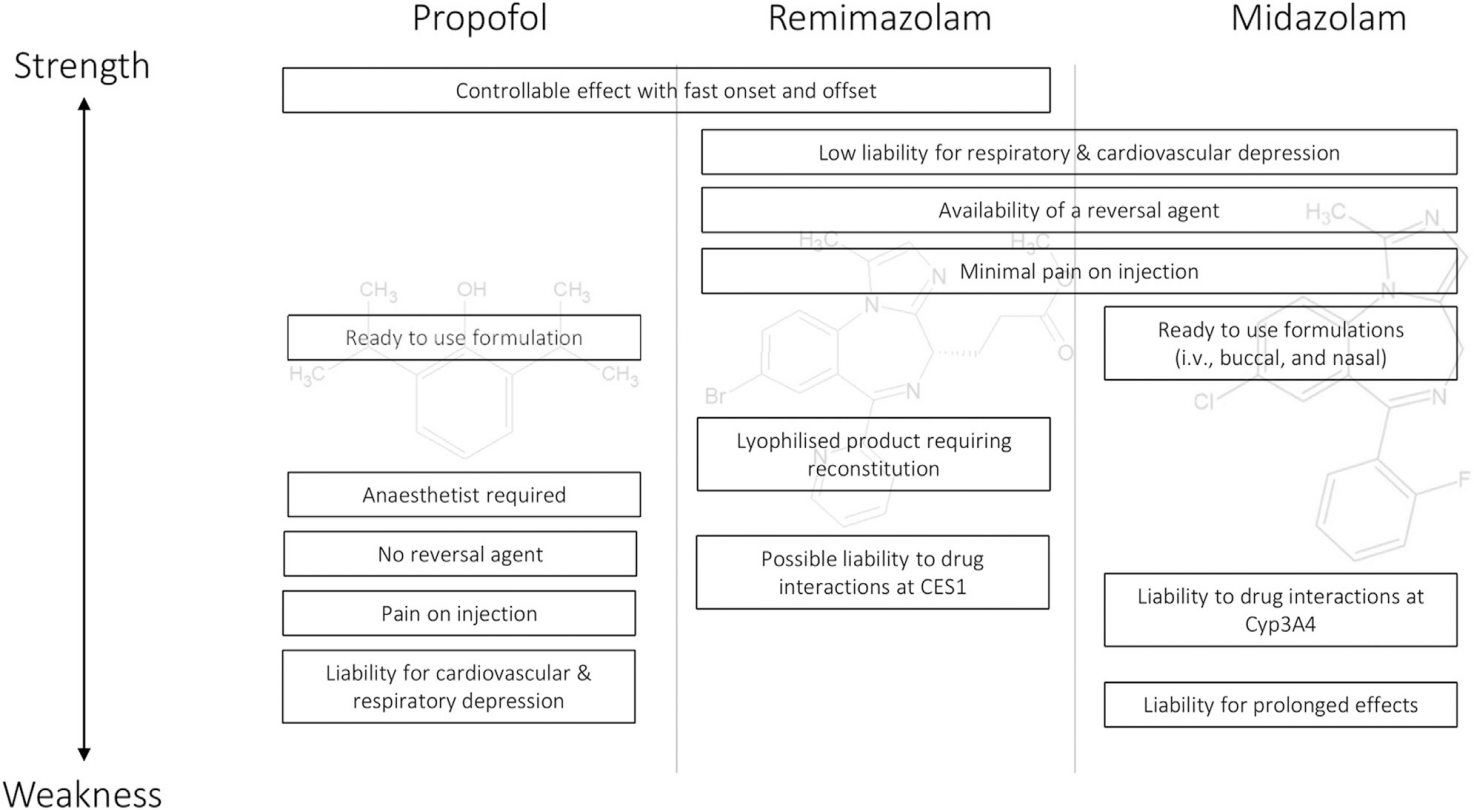
Strength and weakness comparison for the major features of propofol, remimazolam, and midazolam as intravenous sedative/anesthetics.

A comparison of the strengths and weaknesses of remimazolam with midazolam and propofol is shown in Figure 4. The efficacy profile of remimazolam with a predictable and controllable sedative effect and fast onset and offset improves on midazolam. The safety profile of limited cardiovascular and respiratory depression, minimal pain on injection, and the availability of a reversal agent offer advantages over propofol. One complicating feature with the use of remimazolam is that it is a lyophilized product requiring reconstitution rather than the ready-to-use formulations available for propofol and midazolam. The availability of remimazolam is likely to change routine clinical practice in the procedural sedation setting because its efficacy/safety profile will facilitate routine procedures to be conducted rapidly with low liability for deep and/or prolonged sedation. In the anesthesia setting, the compound has the potential to be especially useful in high-risk patients, given the low impact on the cardiovascular and respiratory systems coupled with the availability of a reversal agent.

Comprehension of the complete profile of remimazolam will increase as its use widens, but gaps exist currently. A better understanding is required of the occasional cases of prolonged sedation. A full *in vitro* analysis of the metabolism of remimazolam by various human tissues and characterization of metabolism by specific carboxylesterases and their genetic variants would be a useful addition to the literature. This, together with analysis of the liability for interactions with other drugs metabolized by carboxylesterases, would help to understand potential individual variability in its metabolism and sedative/anesthetic effects. Further clinical data would help to understand better the profile of remimazolam with respect to postoperative complications such as nausea and vomiting. Target engagement analysis employing techniques such as positron emission tomography would provide an enhanced understanding of the interaction of remimazolam with the benzodiazepine site on the GABA_A_ receptor and may facilitate improved PK/PD modeling.

Following the discovery of the compound and early characterisation of the base, the besylate salt form of remimazolam was taken into development. A second development program of the closely related tosylate salt was subsequently initiated in China. Both salt forms have now reached market authorization. No head-to-head comparisons have yet been conducted, but their clinical profiles appear to be virtually identical, as one might expect, given the minor difference. The development challenges of remimazolam (besylate) are worthy of comment. It is unusual for a small company with limited resources to take a compound to the market. While it has taken considerably longer than the industry average to progress to market, this has been achieved by the developer, its predecessors, and its licensees.

The use of intravenous remimazolam is likely to be extended soon to other settings, including sedation in intensive care. Target controlled infusion techniques, employing models of plasma drug concentrations, could enhance its use in general anesthesia. The development of new formulations, e.g., intranasal or inhalation, would enable additional use in situations where intravenous administration is difficult, such as pediatrics, epilepsy, or severe agitation. The availability of this new agent, potentially used in combination with the reversal agent, flumazenil, may also permit further innovations in anesthesia and sedation techniques.
